# Supplementary material for: Protein Kinase D2 drives chylomicron‐mediated lipid transport in the intestine and promotes obesity
Source: EMBO Mol Med. 2021 May 5;13(5):e13548. doi: 10.15252/emmm.202013548 (PMC8103097; doi:10.15252/emmm.202013548)
Supplement: Supplementary file 1 — Appendix [file EMMM-13-e13548-s003.pdf]

Appendix

Table of content

Appendix Table S1. List of Primers

Appendix Table S2. Key Resources

Appendix Table S3. p-values for each experiment

Appendix Table S1. List of Primers

**Gene**	**Forward (5'-3')**	**Reverse (5'-3')**
Pnlip	CTGGGAGCAGTAGCTGGAAG	AGCGGGTGTTGATCTGTGC
Clps	GAACAGTATGCAGTGTAAGAGCA	GCAGATGCCATAGTTGGTGTTG
Cyp7a1	ATCAAAGAGCGCTGTCTGGGT	GCGTTAGATATCCGGCTTCAAAC
Cyp8b1	CTAGGGCCTAAAGGTTCGAGT	GTAGCCGAATAAGCTCAGGAAG
Abcg5	AGGGCCTCACATCAACAGAG	GCTGACGCTGTAGGACACAT
Abcg8	CTGTGGAATGGGACTGTACTTC	GTTGGACTGACCACTGTAGGT
Ehhadh	ATGGCTGAGTATCTGAGGCTG	GGTCCAAACTAGCTTTCTGGAG
Acaa1a	TCTCCAGGACGTGAGGCTAAA	CGCTCAGAAATTGGGCGATG
Cd36	ATGGGCTGTGATCGGAACT	GTCTTCCCAATAAGCATGTCTCC
Bacs	TCTATGGCCTAAAGTTCAGGCG	CTTGCCGCTCTAAAGCATCC
Baat	GGAAACCTGTTAGTTCTCAGGC	GTGGACCCCCATATAGTCTCC
Taat	GCACACGGCCTGAAGATGA	ATTTTTGTAGCAGAGGTACGGG
Csad	CCAGGACGTGTTTGGGATTGT	ACCAGTCTTGACACTGTAGTGA
Asbt	GTCTGTCCCCCAAATGCAACT	CACCCCATAGAAAACATCACCA
Osta	AGGCAGGACTCATATCAAACTTG	TGAGGGCTATGTCCACTGGG
Ostb	AGATGCGGCTCCTTGGAATTA	TGGCTGCTTCTTTCGATTTCTG
Pppara	AACATCGAGTGTCGAATATGTGG	CCGAATAGTTCGCCGAAAGAA
Acadl	TCTTTTCCTCGGAGCATGACA	GACCTCTCTACTCACTTCTCCAG
Fabp4	GGATGG444GTCGACCACAA	TGGAAGTCACGCCTTTCATA
Fabp2	GTGGAAAGTAGACCGGAACGA	CCATCCTGTGTGATTGTCAGTT
Fatp4	ACTGTTCTCCAAGCTAGTGCT	GATGAAGACCCGGATGAAACG
Fatp2	TCGTGGGACTGGTAGATTTTG	CGCGATGTGTTGAAAGAGTTT
Pkd1	GGGGGCATCTCGTTCCAT	GTGCCGAAAAAGCAGGATCTT
Pkd2	GCTGAGACACCTGCACTTCA	GGATCGGCTGATGCCAGTAA
Pkd3	GTCTGTCAAATGTATCTCTGCCA	GGTGAGTATGTGACTCTTCACTG
Mttp1	CTCTTGGCAGTGCTTTTTCTCT	GAGCTTGTATAGCCGCTCATT
Mogat2	TGGGAGCGCAGGTTACAGA	CAGGTGGCATACAGGACGGA
Dgat1	GCGCTACTTCCGAGACTACTT	GGGCCTTATGCCAGGAAACT
Apoa4	GCATCTAGCCCAGGAAACTG	ATGTATGGGGTCAGCTGGAG
Apoa1	GGCACGTATGGCAGCAAGAT	CCAAGGAGGAGGATTCAAACTG
ApoB	AAGCACCTCCGAAAGTACGTG	CTCCAGCTCTACCTTACAGTTGA
Ucp1	AGGCTTCCAGTACCATTAGGT	CTGAGTGAGGCAAAGCTGATTT
Cidea	TGACATTCATGGGATTGCAGAC	GGCCAGTTGTGATGACTAAGAC
Bmp7	ACGGACAGGGCTTCTCCTAC	ATGGTGGTATCGAGGGTGGAA
Adrb3	AGAAACGGCTCTCTGGCTTTG	TGGTTATGGTCTGTAGTCTCGG
Prdm16	CCACCAGCGAGGACTTCAC	GGAGGACTCTCGTAGCTCGAA
Cidec	ATGGACTACGCCATGAAGTCT	CGGTGCTAACACGACAGGG
Pgc1a	AGCGCCGTGTGATTTACGTT	CCGCAGATTTACGGTGCATT
Myh2	AAAGCTCCAAGGACCCTCTT	AGCTCATGACTGCTGAACTCAC
Ckm	CAGCACAGACAGACACTCAGG	GAACTTGTTGTGGGTGTTGC
Mck	GCAAGCACCCCAAGTTTGA	ACCTGTGCCGCGCTTCT
Slc6a8	TGCATATCTCCAAGGTGGCAG	CTACAAACTGGCTGTCCAGA
Slc27a2	TCCTCCAAGATGTGCGGTACT	TAGGTGAGCGTCTCGTCTCG
Ucp3	CTGCACCGCCAGATGAGTTT	ATCATGGCTTGAAATCGGACC
Myh1	TCTGCAGACGGAGTCAGGT	TTGAGTGAATGCCTGTTTGC
**Genotyping**	**Forward (5'-3')**	**Reverse (5'-3')**
$Pkd2^{ki/ki}$	AGTGGCACGTTCCCCTTCAATG	CTTTGCCCAATCCCTTACAGCCT
Vil-Cre/WT	GCCTTCTCCTCTAGGCTCGT	TATAGGGCAGAGCTGGAG GA
Vil-Cre/Tg	GCCTTCTCCTCTAGGCTCGT	AGGCAAATTTTGGTGTACGG
Pkd2-Flox	TGAAGGAAGTGTCTTGGGAGTCCCTGC TGTTTTAATAGC	TGTCTAGGAGGGGACATAACGAACCT GAGGAAACGGATCGGC
**Absolute quantification**	**Forward (5'-3')**	**Reverse (5'-3')**
Pkd1	TTTAACTCCCGTTGGAGCGA	CACTGTTGTTTGGTGGGGAAC
Pkd2	ATGTCACCTACTTTGTGGGCG	TGGGCGCATCTTGGAGGATA
Pkd3	AGGCAGTAACCCACACTGTTT	TCTGCGCCACATCTAGTCCC

Appendix Table S2. Key Resources

REAGENT or RESOURCE	SOURCE	IDENTIFIER
**Antibodies/dilution**		
Rabbit anti-GAPDH 1:20000	Sigma-Aldrich	Cat# G9545, RRID:AB_796208
Rabbit Anti-APOA4/Apo-AIV 1:1000	Abcam	Cat# AB_231660
Mouse anti-apolipoprotein A4 1:1000	Cell Signaling Technology	Cat# 5700, RRID:AB_10859038
Rabbit anti-apolipoproteinB 1:1000	Proteintech	Cat# 20578-1-AP, RRID:AB_10732938
Rabbit anti-apolipoprotein A1 1:1000	Abcam	Cat# ab20453, RRID:AB_445592
Rabbit Anti-MOGAT2 1:1000	Abcam	Cat# ab63156, RRID:AB_956147
Rabbit Anti-DGAT1 1:1000	Abcam	Cat# ab54037, RRID:AB_869453
Rabbit Anti-MTTP 1:1000	Abcam	Cat# ab63467, RRID:AB_10672035
Rabbit Anti-PKD3/PKC,clone (D57E6) 1:1000	Cell Signaling Technology	Cat# 5655, RRID:AB_10695917
Rabbit Anti-Protein Kinase D2, clone (EP1495Y) 1:1000	Abcam	Cat# ab51250, RRID:AB_882058
Rabbit Anti-Protein Kinase D2, clone (D1A7) 1:1000	Cell Signaling Technology	Cat# 8188, RRID:AB_10829368
Rabbit Anti-PKD/PKCμ 1:1000	Cell Signaling Technology	Cat# 2052 RRID:AB_2800149
Rabbit Anti-Phospho-PKD/PKCμ (Ser744/748) 1:1000	Cell Signaling Technology	Cat# 2054 RRID:AB_2172539
Rabbit Anti-Phospho-PKD/PKCμ (Ser916) 1:1000	Cell Signaling Technology	Cat# 2051 RRID:AB_330841
Rabbit Anti-PKD2 (phospho S876), clone (EP1496Y) 1:1000	Abcam	Cat# ab51251 RRID:AB_882060
Rabbit Anti-Phospho-Akt Substrate (RXXS*/T*) (110B7E) 1:1000	Cell Signaling Technology	Cat# 9614 RRID:AB_331810
Mouse anti-β-actin 1:10000	Sigma-Aldrich	Cat# A5441 RRID:AB_476744
Rabbit anti-α-Tubulin 1:5000	Cell Signaling Technology	Cat# 2144 RRID:AB_2210548
Rabbit anti-Pancreatic Lipase 1:2000	Abcam	Cat# ab2287737 RRID:AB_2889168
Goat Anti-Villin, clone (C-19) 1:200	Santa Cruz Biotechnology	Cat# sc-7672, RRID:AB_2215973
Rabbit Anti-Chromogranin A, clone (H-300) 1:200	Santa Cruz Biotechnology	Cat# sc-13090, RRID:AB_2080982
Mouse Anti-E-Cadherin 1:100	BD Biosciences	Cat# 610182, RRID:AB_397581
Gpig Anti-Insulin 1:180	Abcam	Cat# ab7842 RRID:AB_306130
Goat anti-gpig Alexa Flour® 594 1:200	Thermo Fisher Scientific	Cat# A11076 RRID:AB_141930
Donkey anti-mouse Alexa Flour® 555 1:200	Thermo Fisher Scientific	Cat# A-31570, RRID:AB_2536180
Donkey anti-rabbit Alexa Flour® 647 1:200	Thermo Fisher Scientific	Cat# A-31573, RRID:AB_2536183
Donkey anti-Goat IgG Alexa Flour® 555 1:200	Thermo Fisher Scientific	Cat# A-21447, RRID:AB_2535864
**Bacterial and Virus Strains**		
Plasmid: shPkd2. pLKO.1_ MISSION	Sigma-Aldrich	This paper

Plasmid: shPkd3. pLKO.1_ MISSION	Sigma-Aldrich	This paper
Plasmid: pLKO.1-puro scramble Vector Control_MISSION	Sigma-Aldrich	Cat#: SHC001
Plasmid: pLV[shRNA]-Hygro-U6>Scramble_shRNA	Vector Builder	Cat #: Ecoli (VB010000-0006zba)
Plasmid: pLV[shRNA]-Hygro-U6>hPRKD2	Vector Builder	Cat #: Ecoli (VB201013-1031xdu)
Plasmid: psPAX	Addgene	RRID:Addgene_12260
Plasmid: pMD2.G	Addgene	RRID:Addgene_12259
**Chemicals, Peptides, and Recombinant Proteins**		
Recombinant human PKD2 protein	Abcam	Cat#: ab60875
Recombinant Human APOA4/Apo-AIV protein	Abcam	Cat#: ab132653
Pkd inhibitor CRT 0066101	Tocris	Cat#: 4975
Matrigel, Phenol Red-free	Corning	Cat#: 356231
Fluorescein Isothiocyanate Dextran	Sigma-Aldrich	Cat#: 46944
Insulin solution human	Sigma-Aldrich	Cat#: I9278
Triglyceride Reagent	Sigma-Aldrich	Cat#: T2449
Taurocholic acid sodium salt	Sigma-Aldrich	Cat#: T4009
Oleic acid	Sigma-Aldrich	Cat#: O1383
Fatty acids-free BSA	Sigma-Aldrich	Cat#: **A7030**
14C-palmitate	Perkin Elmer	Lot#: 2252435
Fluoromount-G^{TM} with DAPI	Thermo Fisher Scientific	Cat#: 00-4959-52
**Critical Commercial Assays**		
Free Glycerol Determination	Sigma-Aldrich	Cat#: F6428
NEFA-reagents	WAKO	Cat#: 999-34691
Ultra Sensitive Mouse Insulin ELISA Kit	Crystal Chem	Cat#: 90080
**Experimental Models: Cell Lines**		
Caco-2 cells. Not tested for mycoplasma.	ATCC	Cat# HTB-37, RRID:CVCL_0025
HEK293T cells. Last tested for mycoplasma in August 2018.	ATCC	Cat# CRL-3216, RRID:CVCL_0063
**Experimental Models: Organisms/Strains**		
Mouse: *Pkd2*$^{S707A/S711A}$	The Jackson Laboratory	#017285
Mouse *Pkd2* $^{Flox/Flox}$	Lab. Professor Yamasaki	N/A
Mouse: C57BL/6J	The Jackson Laboratory	IMSR Cat# JAX:000664, RRID:IMSR_JAX:000664
Mouse: B6.Cg-Tg(Vil1-cre)1000Gum/J	The Jackson Laboratory	Cat# JAX:021504, RRID:IMSR_JAX:021504
**Oligonucleotides**		
Pkd2 shRNA lentivirus (Sequence 1) CCGGCTTCTACGGCCTTTACGACAACTCGAGTTGTCGTAAAGGCCGTAGAAGTTTTT	Sigma-Aldrich	SHCLNG-NM_016457 TRCN0000001948
Pkd2 shRNA lentivirus (Sequence 2) TCCAGAACAACACGACCAACAGATACTATAAGGAAATTCCG	Vector Builder	Cat #: Ecoli (VB201013-1031xdu)
Pkd3 shRNA lentivirus CCGGCACTTCATTATGGCTCCTAATCTCGAGATTAGGAGCCATAATGAAGTGTTTTTTG	Sigma-Aldrich	SHCLNG-NM_005813 TRCN0000195275
Primers for qPCR and genotyping, see Appendix Table S1		
**Software and Algorithms**		
Illustrator CS6	Adobe	https://www.adobe.com/

Appendix Table S3. p-values for each experiment

Figure		p-value
1A	10	0,0054
	12	0,00059
	14	4,2E-05
	16	0,00035
	18	0,00033
	20	0,00081
	22	0,00012
1B	Fat mass	0,0034
1C	BAT	0,00273
	EpiWAT	0,0008
	SubWAT	0,00092
1D	EpiWAT	0,00066
	SubWAT	0,0062
1F		0,00659
1G	15	0,00681
	30	0,00049
	60	0,00091
	90	0,00063
	120	0,00024
1H	60	0,0098
	90	0,01506
	120	0,00693
1I		0,0451
1J		0,01679
2B		0,00685
2C		0,0052
2D		0,0021
2E		1,5E-05
2F		0,00663
2G		6E-05
2H		8,7E-07
3B	2	0,00816
	3	0,02613
3C	2	0,01402
3D	pPKD upper	0,00883
3E		0,01959
3G	Release	0,003
3H		0,0005
4A		0,01664

Figure		p-value
4B		0,03931
4C	1	0,02639
	2	0,04123
4D	6	0,01279
	8	0,00701
	10	0,00193
	12	0,00187
	14	0,00027
	16	0,00051
4E	Fat mass	0,00661
4F	0	0,00284
	30	0,04949
	60	0,04251
	90	0,03478
4G		0,00706
4H		0,01788
4I	Duodenum	0,0379
4J		0,047
5B		0,02897
5C		0,04228
5D	APOA4	0,00457
5G	50	0,0409
	151-200	0,0163
	201-250	0,0000
	250	0,0030
6A	to 3uM	0,04984
	to 5uM	0,00441
6B	no CRT	0,00644
	shScr -/+ CRT	0,00503
6C		0,11
6D		0,02782
6E	1	0,0283
	2	0,0222
6F		0,03034
6G	7	0,0490
	9	0,0075
	11	0,0092
	13	0,0087
6H	60	0,029

Figure		p-value
6H	90	0,002
6I	0	0,0020
	30	0,0236
	60	0,0467
	90	0,0452
7A	10	0,0049
	12	0,0015
	14	0,0006
	16	0,0006
	18	0,0009
7B	SubWAT	0,0041
	EpiWAT	0,0166
	BAT	0,0219
7C	Fat mass	0,0059
7D	0	0,0288
	15	0,0422
	30	0,0042
	60	0,0070
	90	0,0033
	120	0,0091
7E	15	0,0439
	30	0,0065
	60	0,0366
7G		<0,02
7H		<0,05
EV1A	Prdm16	0,13
	Pgc1a	0,18
	Slc6a8	0,0408
EV1H		0,0035
EV2E	Cyp7a1	0,0459
EV2K	PKD2	0,0109
EV4A	Jejunum	2,916E-06
	Ileum	0,0094
	Duodenum	0,0282
	Large Intest.	0,0137
EV5E	Night	0,0700
	Day	0,1400
EV5F		0,0241

All the p-vales were calculated with a two-tailed Student’s t -test for independent groups. Except for:
4I that was calculated with a non-parametric Mann-Whitney U test
4J which was calculated with a Fisher`s Exact test with adjustments for multiple testing using the Benjamini & Hochberg method
6A-B were calculated by one way ANOVA followed by the post hoc tukey test
