# Supplementary material for: Protein Kinase D2 drives chylomicron‐mediated lipid transport in the intestine and promotes obesity
Source: EMBO Mol Med. 2021 May 5;13(5):e13548. doi: 10.15252/emmm.202013548 (PMC8103097; doi:10.15252/emmm.202013548)
Supplement: Supplementary file 2 — Expanded View Figures PDF [file EMMM-13-e13548-s001.pdf]

Expanded View Figures

Figure EV1. Inactivation of PKD2 does not affect adipose tissue or liver function but increases pancreatic islets size - Related to Fig 1.

A Relative expression of specified genes in SubWAT of *Pkd2*$^{wt/wt}$ and *Pkd2*$^{ki/ki}$ male mice after 22 weeks in HFD. n = 6.
B Representative pictures of H&E staining of liver of indicated male mice in panel (A).
C, D Aspartate aminotransferase (C) and alanine aminotransferase (D) levels in serum of male mice in panel (A). n = 5.
E Glucose-stimulated insulin secretion of *Pkd2*$^{wt/wt}$ and *Pkd2*$^{ki/ki}$ male mice after 8 weeks in HFD. n = 8.
F Representative pictures of immunofluorescent staining for insulin (red) and DAPI (blue) of *Pkd2*$^{wt/wt}$ and *Pkd2*$^{ki/ki}$ male mice. n = 3. For each subject, three different sections of the pancreas were taken and a distance of 50 μm was kept between the sections.
G Mean fluorescence intensity (MFI) for insulin of mice in panel (F). Relative to *Pkd2*$^{wt/wt}$. n = 3. MFI was quantified from all the islets found in each of the three sections per experimental subject.
H Islet area compared to pancreas area of mice in panel (F). Relative to *Pkd2*$^{wt/wt}$. All the islets found in each of the three sections per mouse were quantified.
I Activity of *Pkd2*$^{wt/wt}$ and *Pkd2*$^{ki/ki}$ male mice after 20 weeks in HFD. Expressed as counts per 12 h of intervals. n = 7 for *Pkd2*$^{wt/wt}$ and n = 8 for *Pkd2*$^{ki/ki}$.

Data information: Data presented as average ± SEM, $^*P \leq 0.05$, $^{**}P \leq 0.01$. Significances were assessed by using a two-tailed Student's t-test for independent groups.

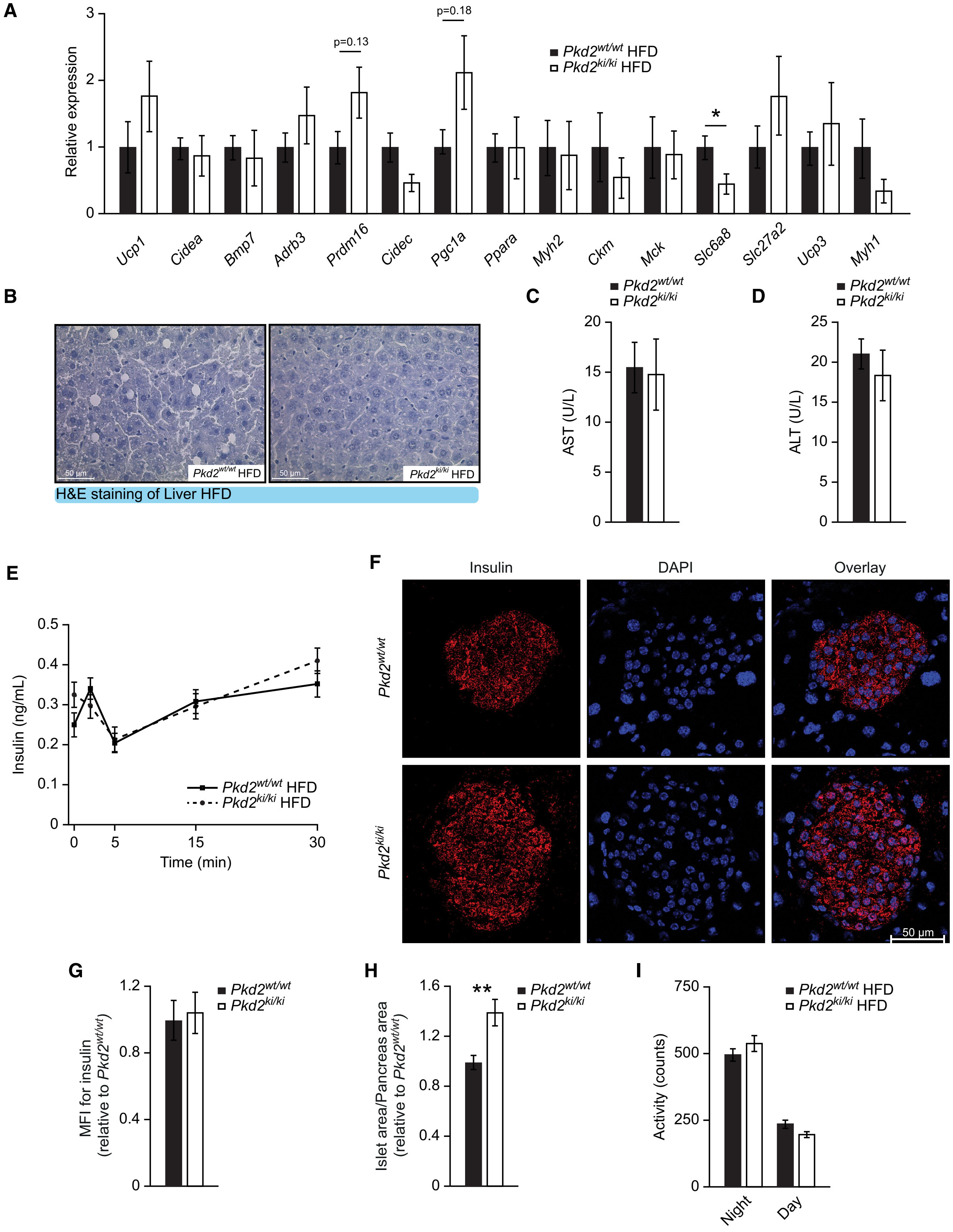

Figure EV1.

© 2021 The Authors

Figure EV2. PKD2 does not regulate the expression of genes implicated in alimentary lipid processing in the intestine or the liver - Related to Fig 2.

A Weight of feces collected from $Pkd2^{wt/wt}$ and $Pkd2^{ki/ki}$ male mice fed normal diet per week. $n = 6$.
B Pictures of feces from $Pkd2^{wt/wt}$ and $Pkd2^{ki/ki}$ male mice under normal diet. $n = 6$.
C Western blot for pancreatic lipase in duodenum of $Pkd2^{wt/wt}$ and $Pkd2^{ki/ki}$ male mice 1 h after olive oil gavage. $n = 6$. The animals were dissected at the indicated time of the day.
D Relative expression of specified genes in pancreas of $Pkd2^{wt/wt}$ and $Pkd2^{ki/ki}$ male mice. $n = 6$.
E Relative expression of genes involved in bile acids production, metabolism, and transport in liver and ileum of male mice with indicated genotypes after 1 week of HFD. $n = 6$.
F Relative expression of genes involved in fatty acid transport in jejunum of indicated male mice. $n = 6$.
G *Pkd1* and *Pkd3* expression levels in duodenum and jejunum of male mice with indicated genotypes. $n = 6$.
H Western blot analysis of protein kinase D1 (PKD1), protein kinase D2 (PKD2), and protein kinase D3 (PKD3) in small intestine of $Pkd2^{wt/wt}$ and $Pkd2^{ki/ki}$ male mice. $n = 3$.
I Quantification of the Western blots from intestine samples in panel (H). $n = 3$.
J Western blot analysis of protein kinase D2 (PKD2) and protein kinase D1 (PKD1) in Caco2 cells expressing *shscramble* or *shPkd2*. $n = 3$.
K Quantification of the Western blots from Caco2 cells presented in panel (J). $n = 3$.

Data information: Data presented as average ± SEM, $*P \leq 0.05$. Significances were assessed by using a two-tailed Student's t-test for independent groups.

© 2021 The Authors

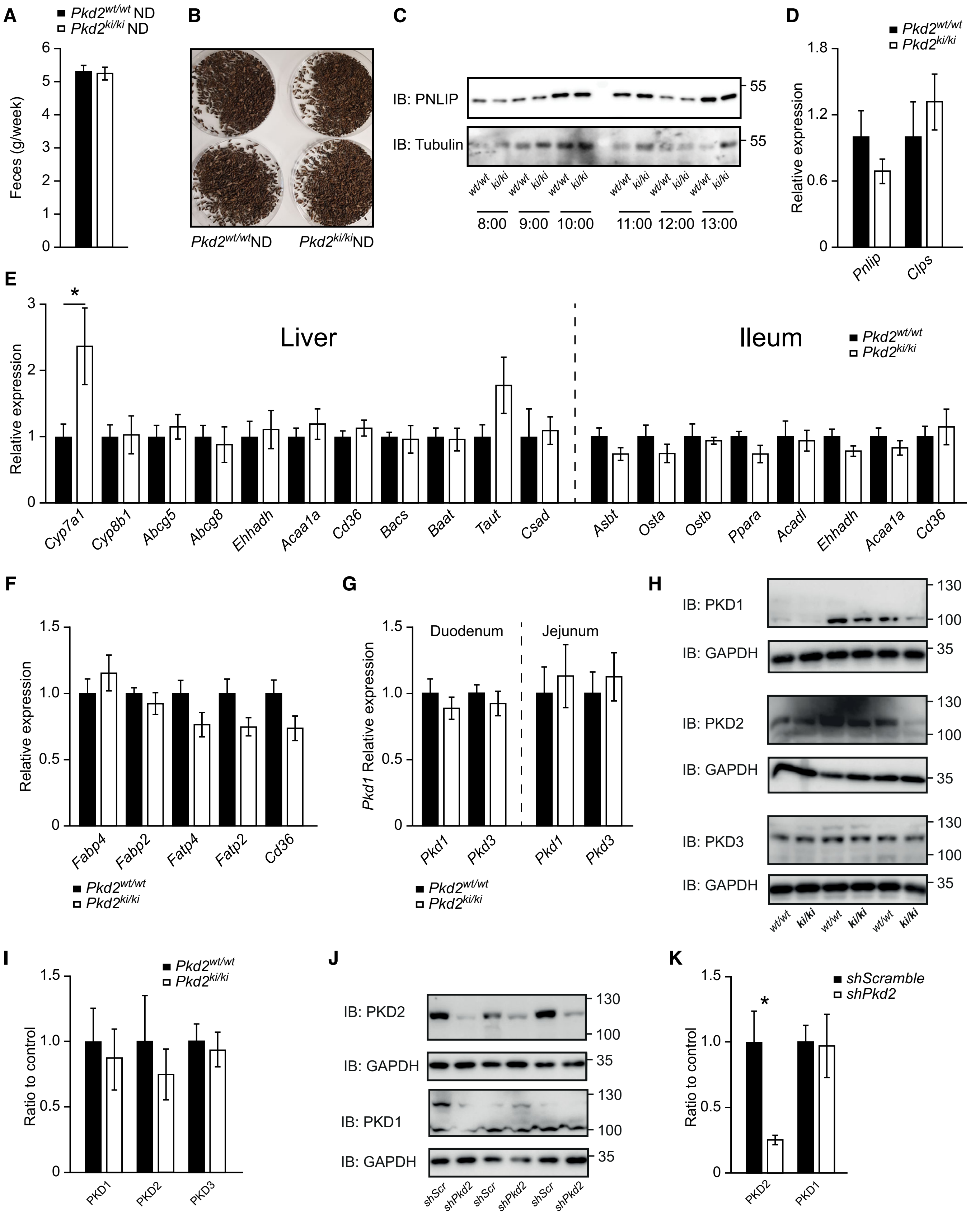

Figure EV2.

© 2021 The Authors

Figure EV3. Inactivation of PKD2 does not affect the architecture, cellularity, or permeability of the intestine - Related to Fig 3.

A Representative pictures of H&E staining of jejunum of *Pkd2*$^{wt/wt}$ and *Pkd2*$^{ki/ki}$ male mice.
B, C Confocal microscopy images of mice-derived organoids stained for E-cadherin, DAPI and villin (B), or chromogranin A (C).
D Dextran concentration in serum after oral gavage of indicated male mice. n = 4
E Length of small intestine of indicated male mice. n = 7 for *Pkd2*$^{wt/wt}$ and n = 8 for *Pkd2*$^{ki/ki}$.
F Western blot of PKD2 in Caco2 cell control (*shScramble*) and depleted from PKD2 by *shRNA* (sequence 1). n = 3.
G Western blot of PKD2 in Caco2 cells expressing *shscramble* or *shPkd2* (sequence 2). Labeled as *shscramble** or *shPkd2**. n = 3.
H Percentage of retention and uptake of ^{14}C-labeled fatty acids in Caco2 cells expressing *shscramble** or *shPkd2** (sequence 2) grown in a Transwell system for 14 days. n = 6.
I Absolute mRNA quantification of *Pkd1*, *Pkd2*, and *Pkd3* in different parts of intestine of C57BL/6 mice. n = 4
J Western blot of PKD3 in Caco2 cell control (*shScramble*) and depleted from PKD3 by *shRNA*. n = 2.
K Percentage of release, uptake, and retention of ^{14}C-labeled fatty acids in Caco2 cells expressing *shscramble* or *shPkd3* grown in a Transwell system for 14 days. n = 5.

Data information: Data presented as average ± SEM. Significances were assessed by using a two-tailed Student's t-test for independent groups.

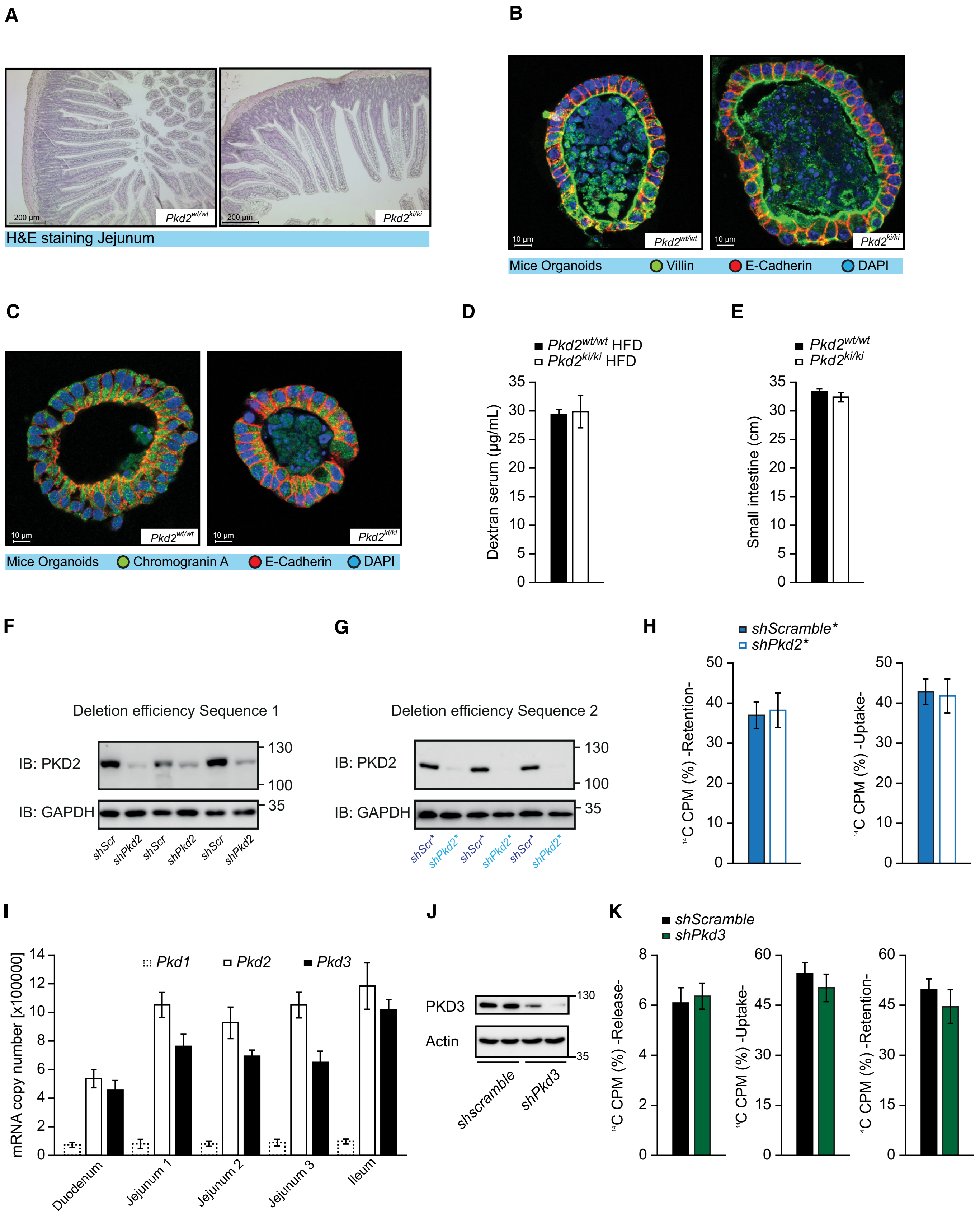

Figure EV3.

 © 2021 The Authors

Figure EV4. Deletion of PKD2 in the intestine does not affect neither food intake nor energy dissipation of the mice, and does not influence hepatic function nor the intestine architecture - Related to Figs 4 and 5.

A Relative expression of *Pkd2* in specified organs of $Pkd2^{flox/flox}$ and $Pkd2^{gut\Delta/\Delta}$ male mice. $n = 4$.
B Western blot of PKD2 in specified organs of $Pkd2^{flox/flox}$ and $Pkd2^{gut\Delta/\Delta}$ mice.
C, D Food intake (C) and energy expenditure (D) of $Pkd2^{flox/flox}$ and $Pkd2^{gut\Delta/\Delta}$ male mice after 13 weeks in HFD. $n = 4$.
E Length of small intestine of indicated male mice. $n = 4$.
F, G Representative H&E pictures of jejunum (F) and liver (G) of male mice with indicated genotypes after 17 weeks in HFD.
H MetaNMDs plots of bacterial composition show specific differences in small intestinal section. $n = 7$.
I Pairwise PERMANOVA indicates no significant differences in compositions of jejunum and ileum. $n = 7$.
J Beta diversity visualized by metaNMDS plots present differences in the microbial composition in duodenum and ileum & jejunum. $n = 7$.
K Relative expression of genes involved in triglyceride and chylomicron synthesis in jejunum of $Pkd2^{wt/wt}$ and $Pkd2^{ki/ki}$ male mice after 1 week in HFD. $n = 7$.

Data information: Data presented as average ± SEM, *$P \le 0.05$, **$P \le 0.01$, ***$P \le 0.001$. Significances in panels (A–E) were assessed by using a two-tailed Student's *t*-test for independent groups. In panels (I–K), statistical testing was performed using PERMANOVA with corrections for multiple testing using the Benjamini and Hochberg method.

© 2021 The Authors

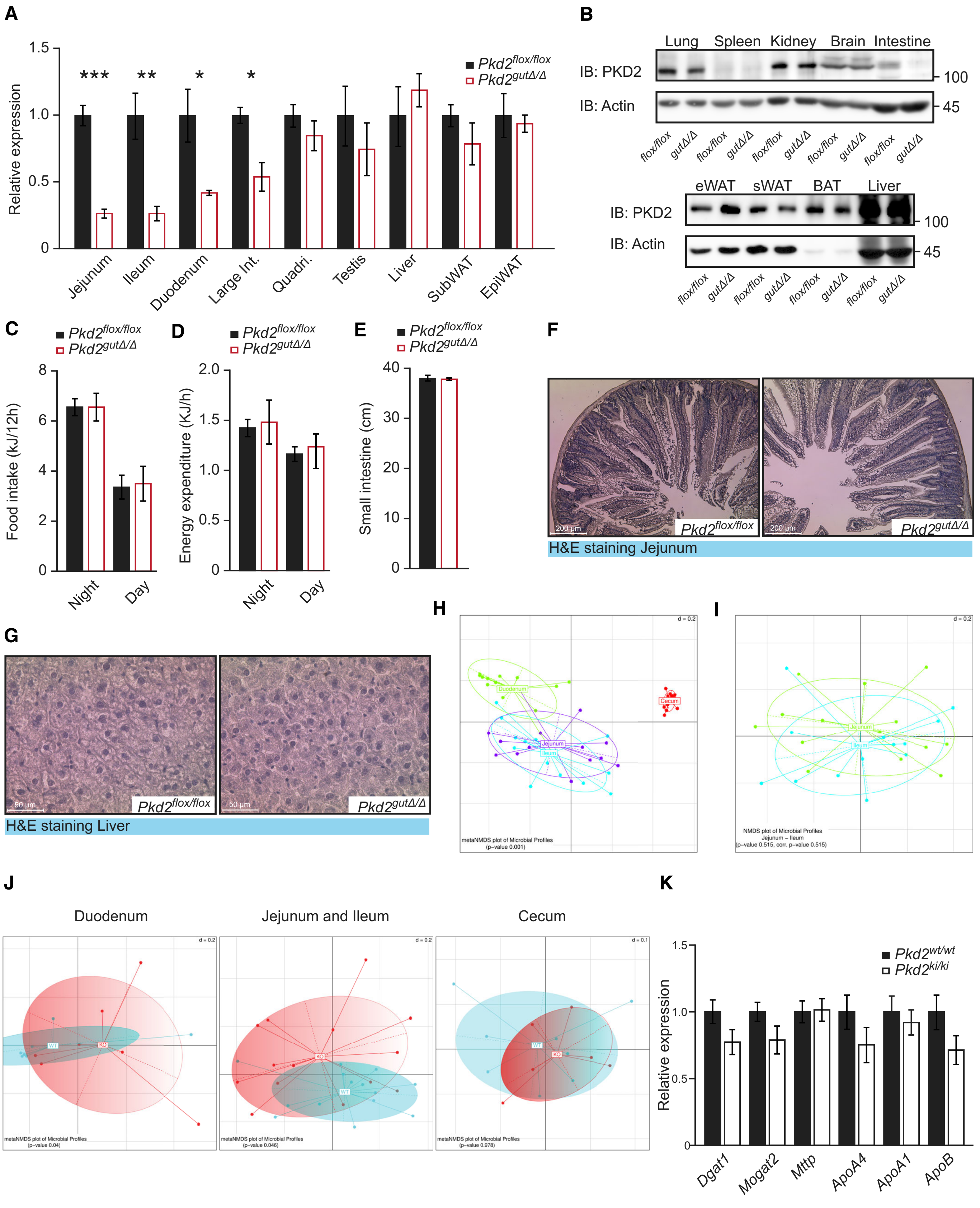

Figure EV4.

© 2021 The Authors

Figure EV5. Oral dosing of PKD inhibitor (CRT0066101, 10 mg/kg of body weight) does not affect PKD activity in the liver or adipose tissue - Related to Figs 6 and 7.

A Western blot and quantification of phosphorylated PKD (S916/S876) in liver of male C57BL/6 mice in HFD which received CRT0066101 or water (control) for 13 weeks. $n = 3$.
B Western blot and quantification of phosphorylated PKD (S916/S876) in EpiWAT of male C57BL/6 mice fed HFD which received CRT0066101 or water (control) for 13 weeks. $n = 3$.
C–E Energy intake (C), energy expenditure (D), and activity (E) of male C57BL/6 mice which received CRT0066101 or water (control). Measurements after 7 weeks of treatment and HFD. $n = 4$.
F Insulin levels of male C57BL/6 mice fed with HFD which received CRT0066101 or water (control) for 13 weeks. Samples taken after 4 h fasting and 1-h refeeding. $n = 6$ for control and $n = 5$ for CRT0066101.
G Alanine aminotransferase (ALT) and aspartate aminotransferase (AST) levels in serum of male C57BL/6 mice fed HFD which received CRT0066101 or water (control) for 13 weeks. $n = 6$ for control and $n = 5$ for CRT0066101.
H Triglyceride content in the liver of male C57BL/6 mice fed HFD which received CRT0066101 or water (control) for 13 weeks. Relative to control. $n = 6$ for control and $n = 5$ for CRT0066101.
I Representative H&E pictures of brown adipose tissue (BAT) of male C57BL/6 mice in HFD which received CRT0066101 or water (control) for 13 weeks.
J Intestinal permeability measured by dextran gavage of mice in Fig 7A. $n = 8$.
K Anthropometric, metabolic, and biochemical data from human female patients in Fig 7F–I. $n = 7$.

Data information: Data presented as average ± SEM, $*P \leq 0.05$. Significances were assessed by using a two-tailed Student's t-test for independent groups.

© 2021 The Authors

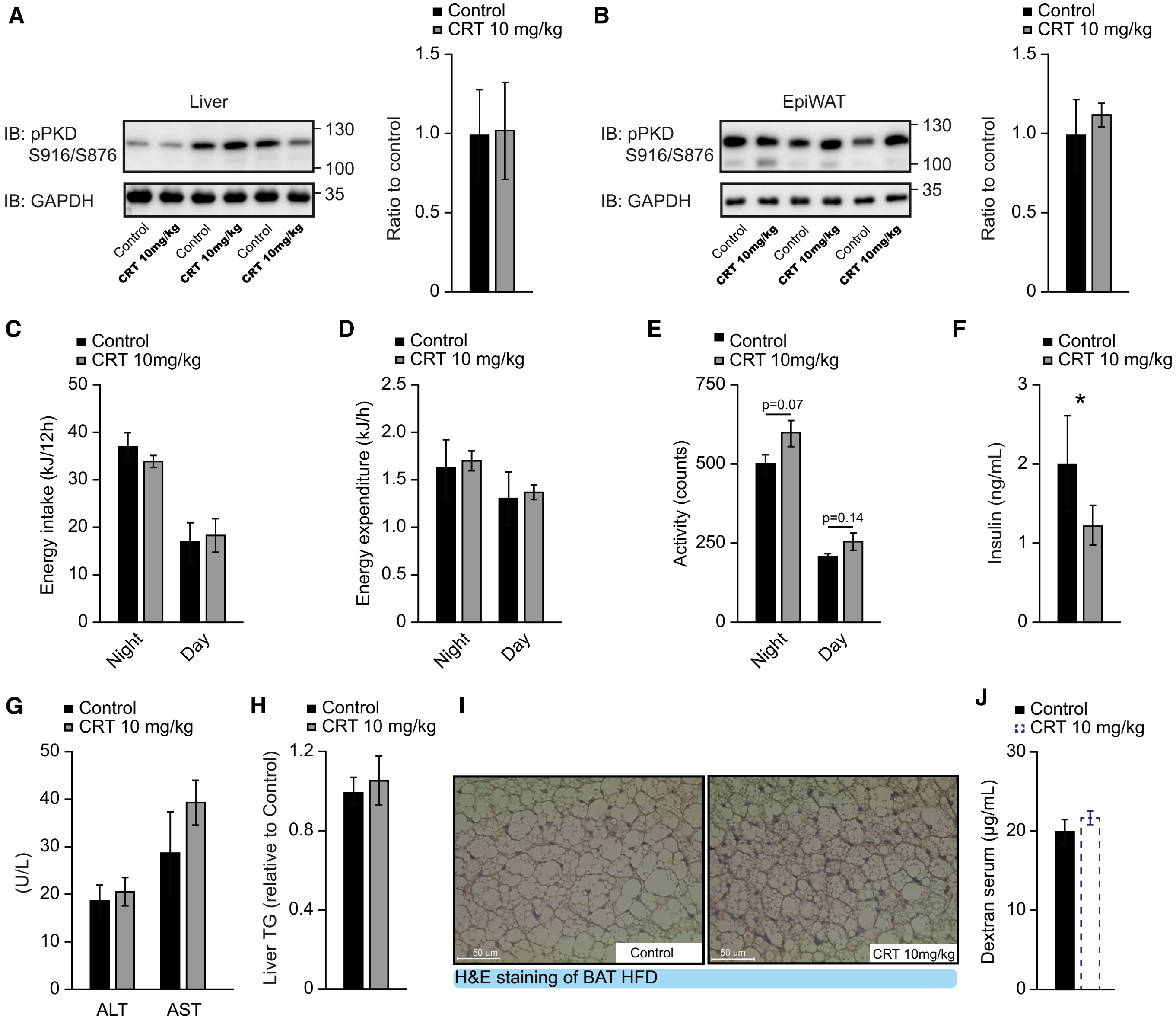

K

Patient	Weight (kg)	Height (cm)	BMI (kg/m^2)	T2D	Fasting glucose (mg/dL)	Cholesterol (mg/dL)	LDL (mg/dL)	HDL (mg/dL)	HbA1c (%)	Triglycerides (mg/dL)	pPKD/GAPDH (relative to patient 1)
1	158	175	51.7	No	90	181	107	58	5.3	79	1
2	128	161	49.6	No	99	221	127	70	5.6	122	0.52
3	112	162	42.7	No	97	210	137	46	6.1	136	0.85
4[a]	127	168	45	No	92	174	93	53	5.3	138	0.86
5	158	171	54	No	85	149	80	40	6.1	143	1.05
6[b]	137	170	47.4	Yes	86	269	181	40	6.2	238	1.28
7[b]	122	163	45.9	Yes	95	165	63	44	6.8	291	1.84

[a]Medicated with metformin for PCOS [b]Medicated with metformin for T2D

Figure EV5.

© 2021 The Authors
