# Supplementary figures and images for: Protein Kinase D2 drives chylomicron‐mediated lipid transport in the intestine and promotes obesity
Source: EMBO Mol Med. 2021 May 5;13(5):e13548. doi: 10.15252/emmm.202013548 (PMC8103097; doi:10.15252/emmm.202013548)

# Source data Fig 3

Fig 3A

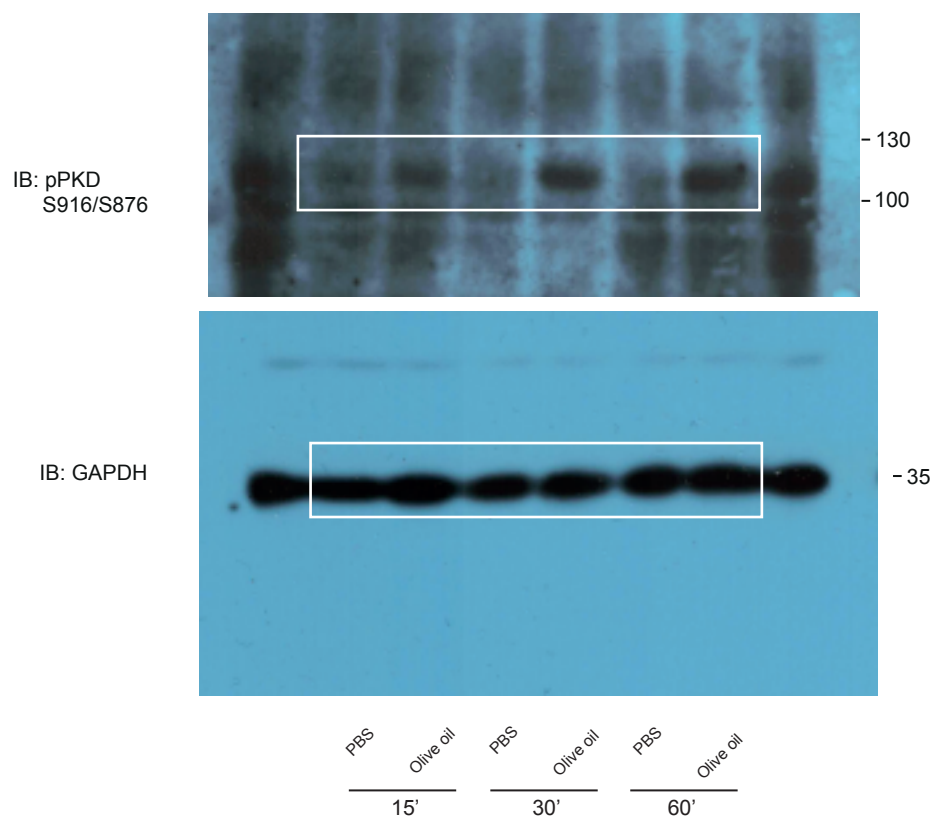

Fig 3D up

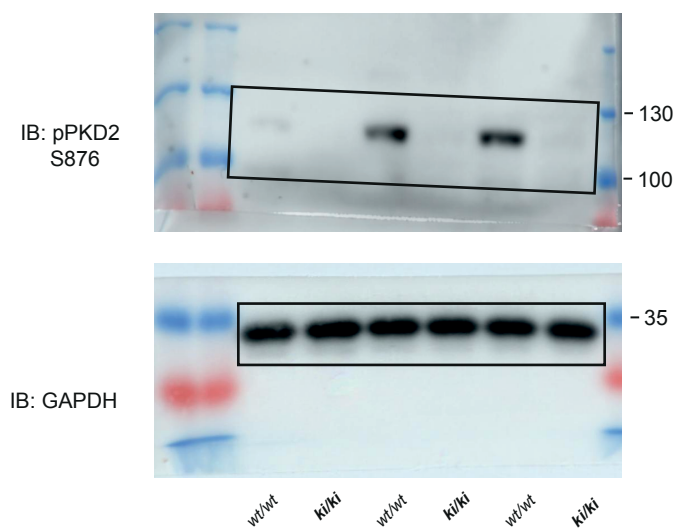

Fig 3D low

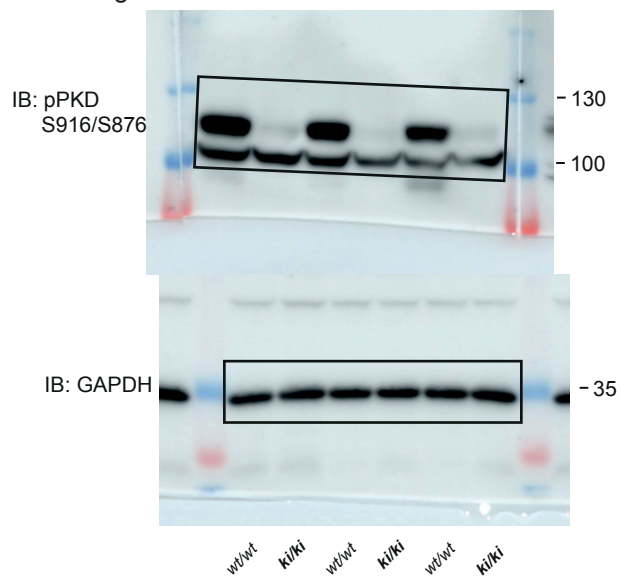

Supplement: Supplementary file 4 — Source Data for Figure 3 [file EMMM-13-e13548-s002.pdf]

Source data Fig 4

Fig 4A

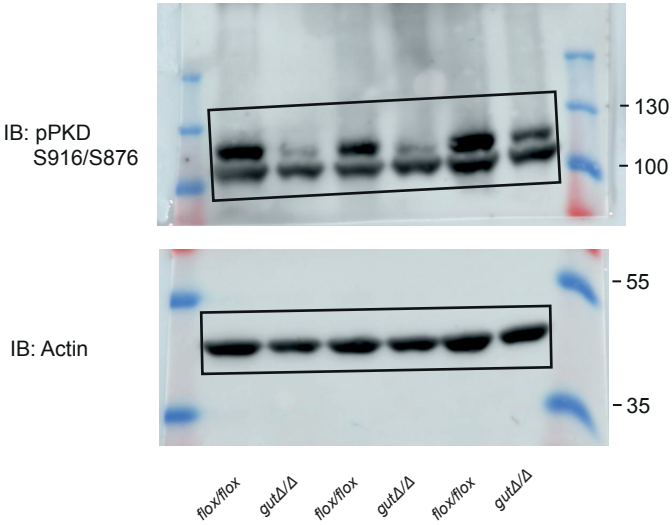

Fig 4B

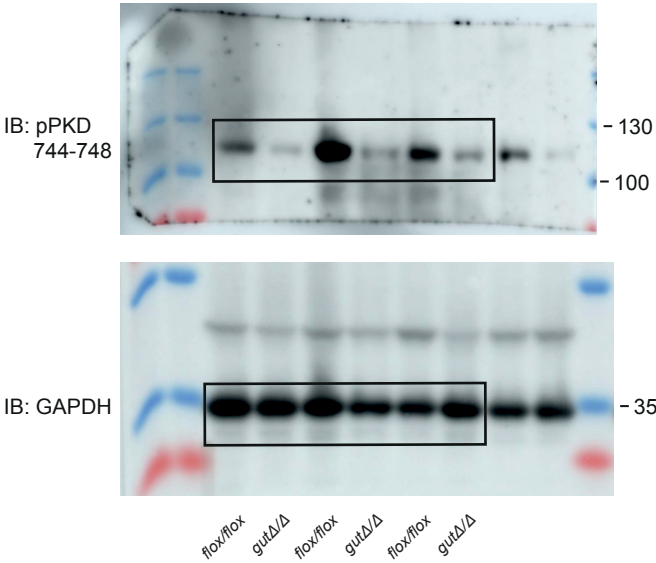

Supplement: Supplementary file 5 — Source Data for Figure 4 [file EMMM-13-e13548-s004.pdf]

Source data Fig 5

Fig 5A

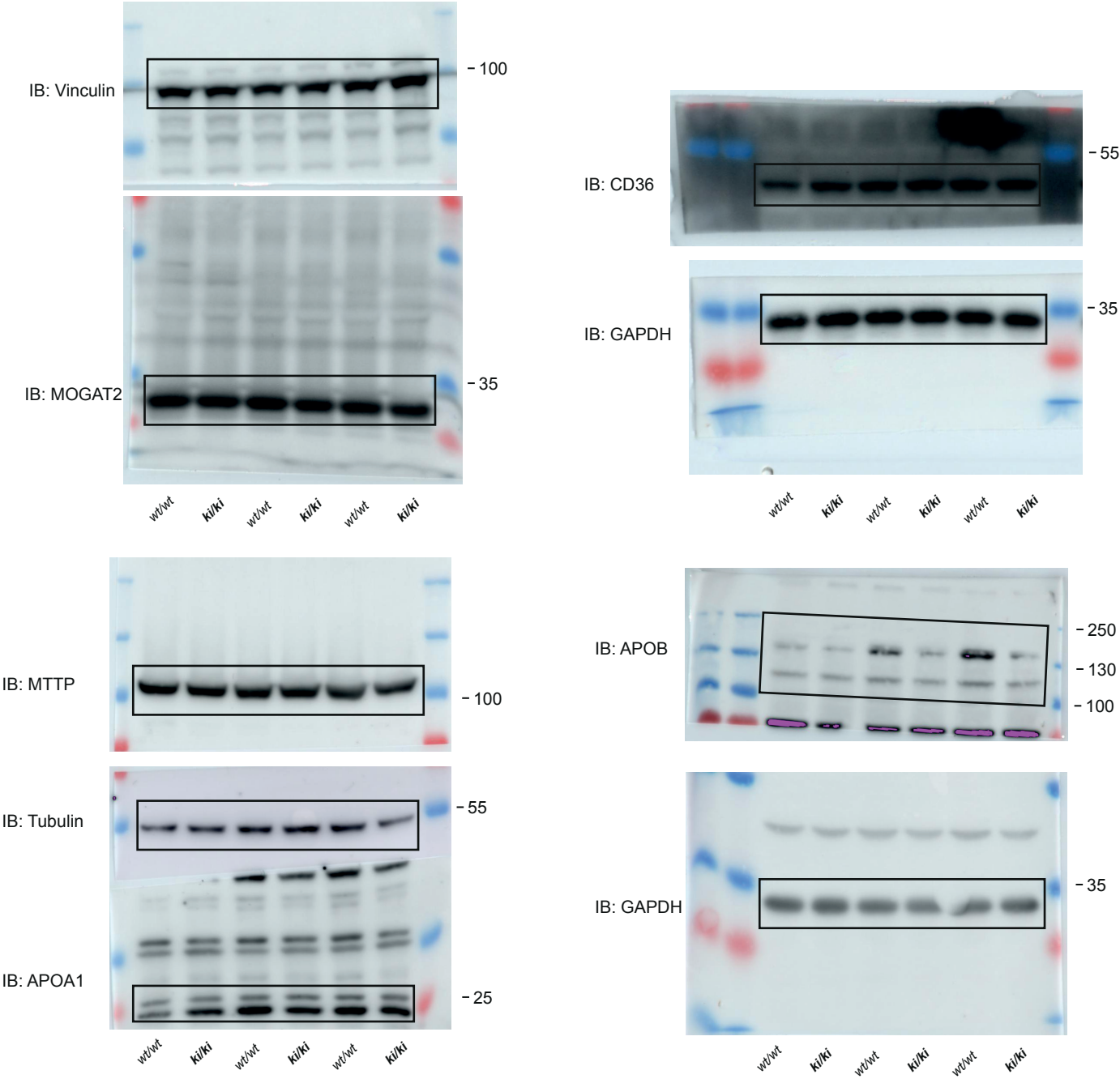

Source data Fig 5

Fig 5B

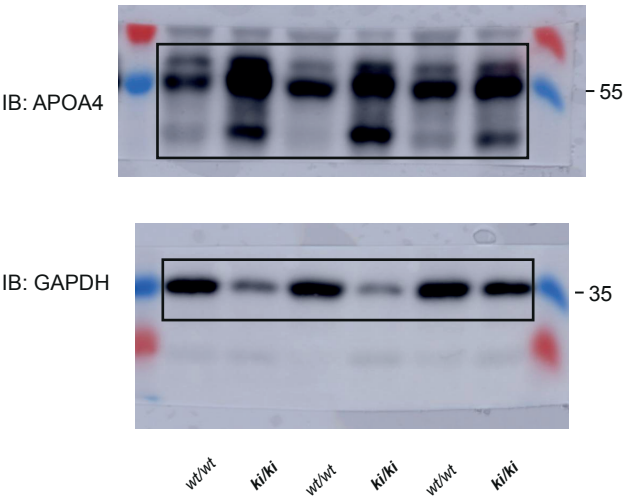

Fig 5C

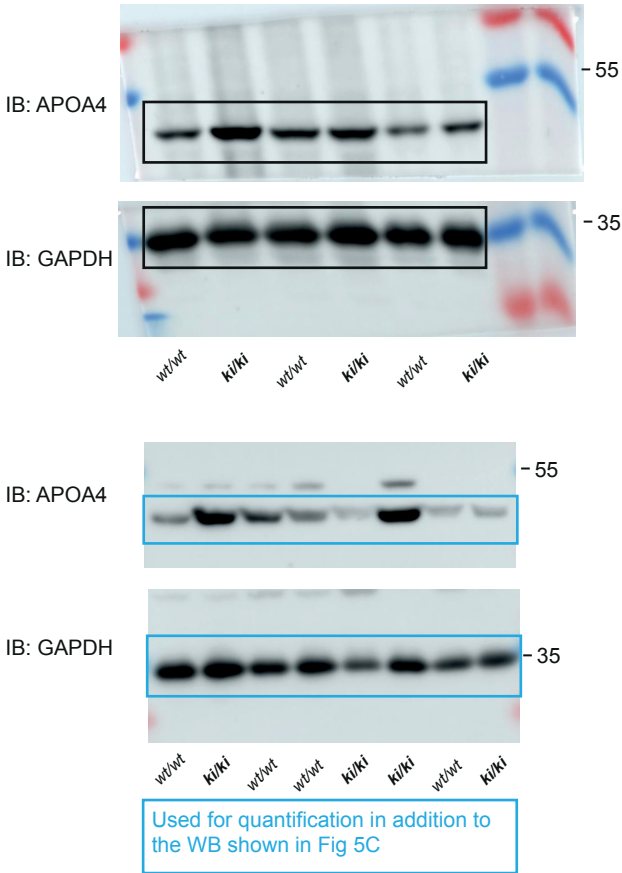

Fig 5D

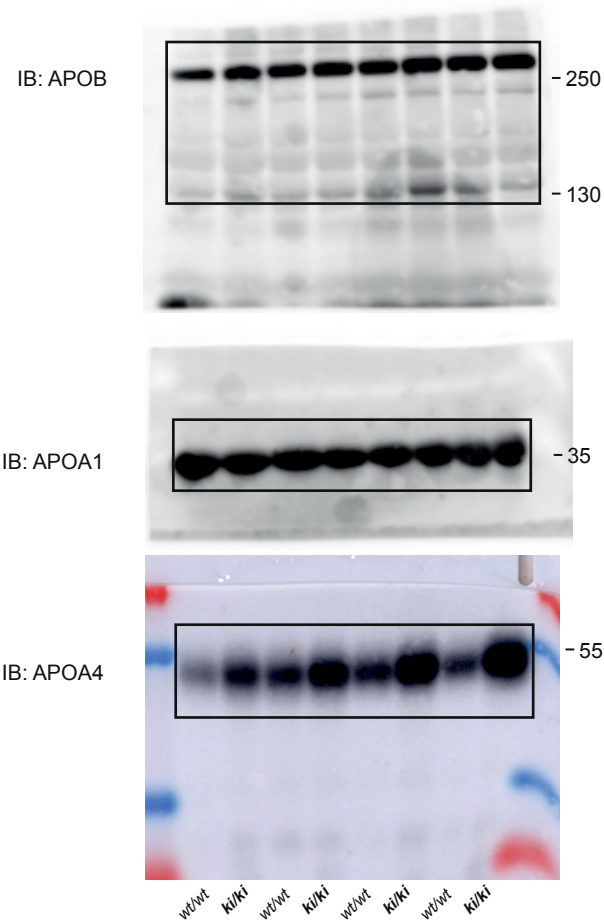

Fig 5E

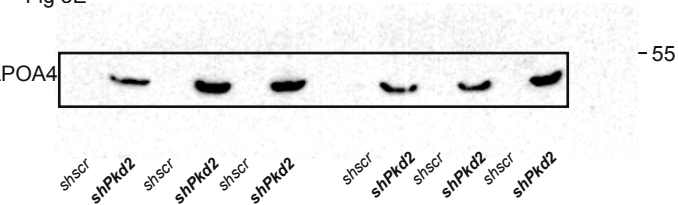

Fig 5H

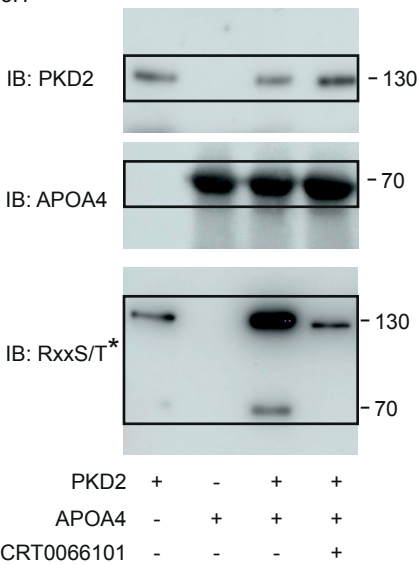

Supplement: Supplementary file 6 — Source Data for Figure 5 [file EMMM-13-e13548-s006.pdf]

Source data Fig 6

Fig 6C

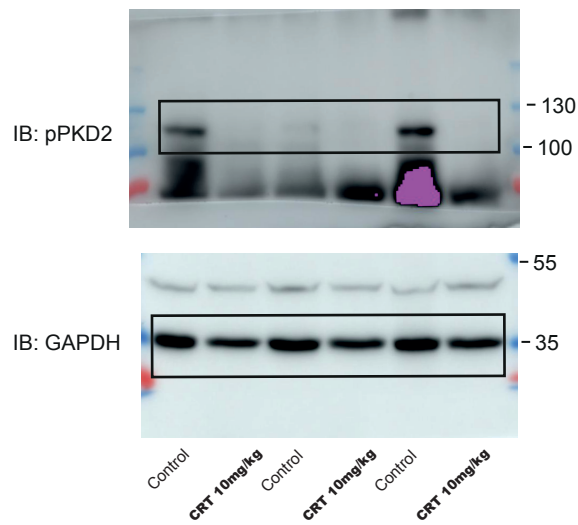

Fig 6F

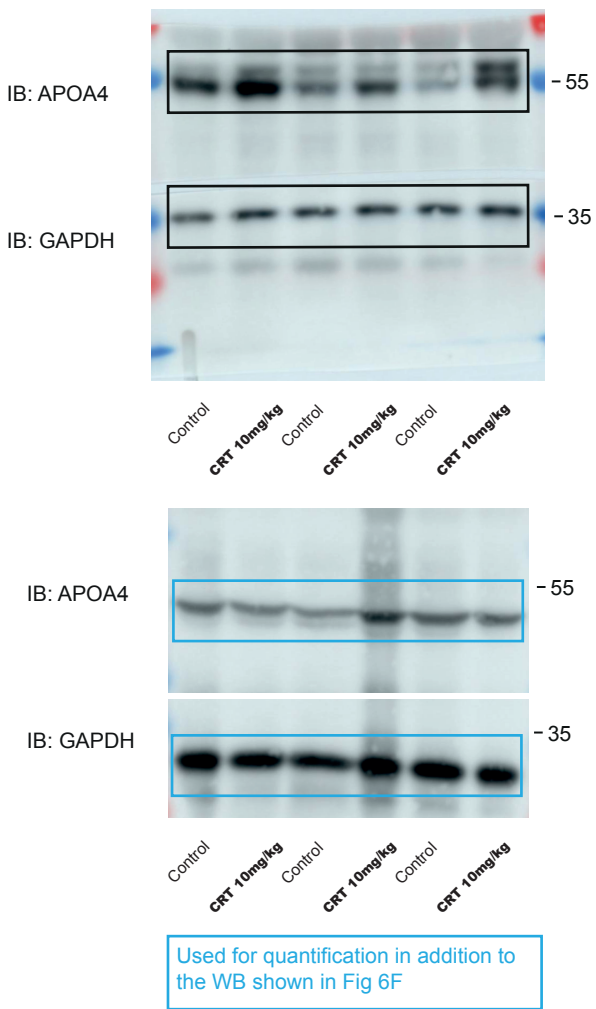

Supplement: Supplementary file 7 — Source Data for Figure 6 [file EMMM-13-e13548-s007.pdf]

## Source data Fig 7

Fig 7F

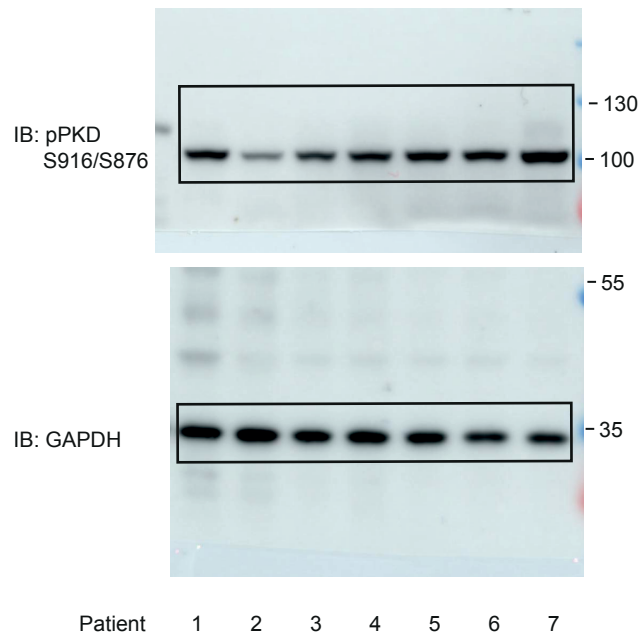

Supplement: Supplementary file 8 — Source Data for Figure 7 [file EMMM-13-e13548-s005.pdf]
